# Advanced Graphene-Based Transparent Conductive Electrodes for Photovoltaic Applications

**DOI:** 10.3390/mi10060402

**Published:** 2019-06-17

**Authors:** Susana Fernández, Alberto Boscá, Jorge Pedrós, Andrea Inés, Montserrat Fernández, Israel Arnedo, José Pablo González, Marina de la Cruz, David Sanz, Antonio Molinero, Rajveer Singh Fandan, María Ángela Pampillón, Fernando Calle, José Javier Gandía, Julio Cárabe, Javier Martínez

**Affiliations:** 1 CIEMAT, División de Energías Renovables, Avda. Complutense 40, 28040 Madrid, Spain; JosePablo.Gonzalez@ciemat.es (J.P.G.); marina.cruz@ciemat.es (M.d.l.C.); jj.gandia@ciemat.es (J.J.G.); julio.carabe@ciemat.es (J.C.); 2 Instituto de Sistemas Optoelectrónicos y Microtecnología, Universidad Politécnica de Madrid, Avda. Complutense 30, 28040 Madrid, Spain; alberto.bosca@upm.es (A.B.); j.pedros@upm.es (J.P.); rajveer.fandan@upm.es (R.S.F.); m.pampillon@upm.es (M.Á.P.); fernando.calle@upm.es (F.C.); 3 Departamento de Ingeniería Electrónica, E.T.S.I de Telecomunicación, Universidad Politécnica de Madrid, Avda. Complutense 30, 28040 Madrid, Spain; 4 Das-Nano, Polígono Industrial Talluntxe, Calle M-10, Tajonar, 31192 Navarra, Spain; aines@das-nano.com (A.I.); mfernandez@das-nano.com (M.F.); iarnedo@das-nano.com (I.A.); 5 Departamento Ingeniería Eléctrica, Electrónica y de Comunicación, Universidad Pública de Navarra, Campus Arrosadía, 31006 Pamplona, Spain; 6 CIEMAT, Departamento de Electrónica, Avda. Complutense 40, 28040 Madrid, Spain; dasago93@gmail.com (D.S.); antonio.molinero@ciemat.es (A.M.); 7 Departamento de Ciencia de Materiales, E.T.S.I de Caminos, Canales y Puertos, Universidad Politécnica de Madrid, C/ Profesor Aranguren s/n, 28040 Madrid, Spain

**Keywords:** graphene, transparent electrodes, silicon heterojunction solar devices

## Abstract

New architectures of transparent conductive electrodes (TCEs) incorporating graphene monolayers in different configurations have been explored with the aim to improve the performance of silicon-heterojunction (SHJ) cell front transparent contacts. In SHJ technology, front electrodes play an important additional role as anti-reflectance (AR) coatings. In this work, different transparent-conductive-oxide (TCO) thin films have been combined with graphene monolayers in different configurations, yielding advanced transparent electrodes specifically designed to minimize surface reflection over a wide range of wavelengths and angles of incidence and to improve electrical performance. A preliminary analysis reveals a strong dependence of the optoelectronic properties of the TCEs on (i) the order in which the different thin films are deposited or the graphene is transferred and (ii) the specific TCO material used. The results shows a clear electrical improvement when three graphene monolayers are placed on top on 80-nm-thick ITO thin film. This optimum TCE presents sheet resistances as low as 55 Ω/sq and an average conductance as high as 13.12 mS. In addition, the spectral reflectance of this TCE also shows an important reduction in its weighted reflectance value of 2–3%. Hence, the work undergone so far clearly suggests the possibility to noticeably improve transparent electrodes with this approach and therefore to further enhance silicon-heterojunction cell performance. These results achieved so far clearly open the possibility to noticeably improve TCEs and therefore to further enhance SHJ contact-technology performance.

## 1. Introduction

Graphene has been regarded as a promising candidate for the new emerging generation of transparent electrodes in several applications such as displays, touch screens and/or solar cells [1,2]. Its unique mechanical, electrical and optical properties make it an industrially and economically relevant material for the next years [3,4,5]. One of the main application areas of transparent electrodes is photovoltaics (PV) and graphene possesses most, if not all of the properties required for it to offer innovative solutions in the field: high optical transmittance, exceptional electronic transport, outstanding mechanical strength and environmental stability [6].

Significant effort has been devoted to improve the overall performance of PV devices using graphene. Thus, this material has been reported to play diverse positive roles acting as electrode, active layer, interfacial layer or electron acceptor in photovoltaic cells [7,8,9,10,11,12]. However, research on solar cells containing graphene in their structure is still at laboratory scale, owing to both a lack in the ability to produce large-sized graphene sheets and a relatively low reproducibility of its properties.

PV market is dominated by crystalline-silicon technology that requires exhaustive technological solutions to achieve thinner and cheaper wafers. In this sense, silicon-heterojunction (SHJ) technology is emerging as a reliable low-temperature and high-efficiency solution, where new architectures of transparent conductive electrodes (TCEs) to generate and extract the current in a more efficient way are being required [7]. In that device, the role of the transparent conductive oxide (TCO) is crucial for both efficient carrier collection and transport properties. Hence, a suitable optical thickness and refractive index of the TCO must be matched with the whole structure. Highly degenerated n-type indium tin oxide (ITO) is stablished as the most potential TCO to be used in solar cells due to its excellent transparency and conducting properties. On the other hand, aluminum-doped zinc oxide (AZO) is also been widely used as potential substitute of ITO because of the indium scarcity. 

Regarding the prominent and unique properties of graphene, several approaches incorporating it have proven to be especially interesting. In particular, hybrid concepts combining graphene with metal grids [13] or nanowire networks [14,15] have already been demonstrated as high-quality flexible TCEs, where the graphene replaces the TCOs typically used. However, in the SHJ technology the TCO film plays also an important role as anti-reflective coating. Therefore, hybrid graphene/TCO structures capable of enhancing the performance of the SHJ technology are highly desirable. 

In the present work, new architectures of TCOs and graphene electrodes incorporating one, two and three atomic graphene layers in different configurations have been fabricated in order to evaluate the optoelectronic properties of the resulting structures. The key parameters for choosing the most appropriated and reliable combination of layers for the electrode have been determined.

## 2. Materials and Methods 

### 2.1. CVD Graphene Synthesis and Automatic Graphene Transfer

A commercial copper foil was used as a catalyst for the chemical vapor deposition (CVD) of the graphene (25-µm-thick Cu foil, 99.8% purity, Alfa Aesar, Kandel, Germany). This foil was cut into 5 cm × 6 cm rectangles and subsequently dipped in 55 wt.% phosphoric acid and then electro-polished for 10 min. After that, a hot peroxide treatment at 80 °C for 10 min was carried out.

A cold-wall CVD reactor (Black Magic Pro, Aixtron Ltd., Cambridge, UK) for 4-inch-diameter samples was used for the synthesis of graphene monolayer. The system has two heaters, a bottom one under the sample and one at the top heating the gases from the showerhead and also capable of radiatively heating non-flat or thick samples [16]. All the process gases (argon (Ar), hydrogen (H_2_), and methane (CH_4_)) connected to the CVD chamber were flushed before the growth. After that, the temperature was raised to the synthesis temperature (1050 °C) under Ar atmosphere. Once the temperature was stabilized, a mixture of 0.7% CH_4_/31% H_2_/68.3% Ar (in volume) was introduced for 15 min at 50 mbar pressure. 

The synthesized graphene on the Cu foil was transferred to the different substrates used throughout the study using a home-built system designed to automatically transfer graphene to arbitrary substrates [17]. This system allows the all-fluidic manipulation of the graphene to avoid mechanical damage, strain, and contamination of the graphene, leading to improved material quality and efficiency. It is composed of several modules that control the temperature, the liquid flow, and the overall system state. A microcontroller is used as the real-time control. A polytetrafluoroethylene tube encloses the graphene sample during the whole process, wherein the combination of the capillary action and the electrostatic repulsion between the graphene and its container ensures a centered sample on top of the target substrate. The starting liquid used in the tube is a Cu etchant (ammonium persulfate ((NH_4_)_2_S_2_O_8_) 0.3 M), which is gradually changed into deionized water (DIW) for the final steps. 

### 2.2. Transparent Conductive Oxide Fabrication

A commercial UNIVEX 450B sputtering system from Leybold, Cologne, Germany equipped with four magnetron sources was used for the fabrication of TCO thin films. This system has a confocal geometry where the guns are placed at around 0.15 m from the substrate centre. The base pressure of the process chamber is 1 × 10^−5^ Pa. The purity of Ar gas used for the sputtering processes is 99.999%, and its flux is controlled with a mass-flow controller. In this work, aluminum-doped zinc oxide (AZO) and indium tin oxide (ITO) were chosen as TCO materials. The 4-inch commercial ceramic targets of ZnO:Al_2_O_3_ (98/2 wt.%, Neyco) and In_2_O_3_:SnO_2_ (90/10 wt.%, Neyco) for AZO and ITO deposition respectively were sputtered by radio-frequency (RF) and direct-current (DC) power for AZO and ITO, respectively.

### 2.3. Transparent Conductive Electrodes Based on Graphene

The TCE structures were deposited on 4-inch polished resistive float zone <100> silicon wafers (resistivity > 10^4^ Ω·cm). The native silicon oxide (SiO_x_) was removed from the silicon-wafer surface by chemically etching with a dilute (2%) hydrofluoric acid. After that, the wafer was rinsed in DIW and finally, dried by blowing nitrogen over it. On the other hand, glass substrates were also used to determine the TCE optical transparency. The glass substrates were ultrasonically cleaned using an special detergent that is a phosphate free surface active cleaning agent radioactive decontaminant from DECON laboratories , rinsed in DIW, and finally, immersed for 2 min in isopropyl alcohol and dried by blowing nitrogen over it.

**Figure 1:**
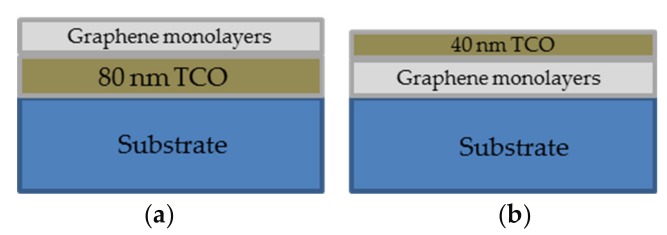
TCE configurations under study (**a**) configuration 1, and (**b**) configuration 2.

Two different configurations were studied for the graphene-based TCE. In Figure 1a, the graphene monolayers (GML) were transferred onto the already TCO-coated substrate. In Figure 1b, the TCO layer was sputtered on top of the GML previously transferred onto the substrate. In all cases, the TCO optical thickness was chosen to be an odd number of quarter wavelengths (*λ*/4) in order to lead to the destructive interference of the beams reflected from the silicon and TCO surfaces, thus acting as an anti-reflectance (AR) coating. The number of GML was chosen in order to obtain a TCE with a good conductivity and low optical losses.

In the particular case of the configuration 1, an Ar plasma bias etching on the substrate surface was carried out at 120 W under 0.5 Pa for 5 min without intentional heating prior to the sputtering deposition. This process was used to improve TCO thin-film adherence. After that, the substrate was rotated at 20 rpm, and heated at 190 °C in the holder system. Regardless the TCO material deposited, the gas flow rate and working pressure were maintained constant at 5 sccm and 0.18 Pa, respectively. The samples were sputtered at the constant values of 75 W DC power and 250 W RF power applied to ITO and AZO targets, respectively. In the case of configuration 2, no bias was applied and softer sputtering conditions were used to avoid the possible damage to GML structure, that is, at room temperature and 25 W DC power and 150 W RF applied to ITO and AZO targets, respectively.

### 2.4. Characterization Techniques

The structural quality of the graphene in the TCE was evaluated from Raman measurements. A WITec Alpha 300AR, Ulm, Germany, confocal microscope was used for the Raman spectroscopy of the samples. Raman spectra were obtained in backscattering geometry using a ×100 objective lens (numerical aperture NA = 0.95) in ambient conditions. A 532 nm wavelength laser set to a power of 3 mW was used as excitation source. 

**Figure 2:**
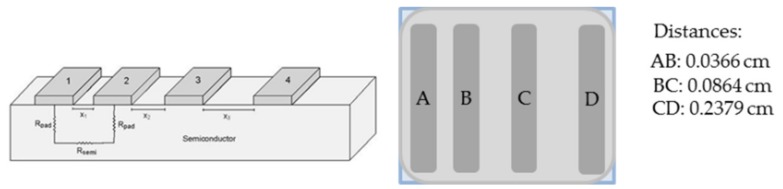
Schematic of the transmission line model (TLM) structure used to measure the electrical parameters of the fabricated TCEs.

Four metal coplanar parallel electrodes unequally spaced, pictured in Figure 2, were deposited by thermal evaporation onto the GML previously transferred onto 1 × 1 cm^2^ quartz substrates. 

The metal combination tested to have a low contact resistance was Ti (50 nm)/Ag (500 nm). Transmission-line-model (TLM) measurements were done by testing 4-point electrical resistance between all the possible electrode pairs with two contacts on each electrode. Contacts were made by means of 4 micro-positioners, a power supply was used to bias the samples, an electrometer measured currents and a voltmeter provided voltages. These TLM measurements were used to determine the actual sheet resistance of the hybrid TCEs developed (*R_TCE_*) and to compare them to the theoretical values expected corresponding to an electric circuit with two parallel electric resistances, as described by the following Equation:

(1) $$\frac{1}{R_{TCE}}=\frac{1}{R_{TCO}}+\frac{1}{R_{GG}}$$

where *R_TCO_* is the sheet resistance of the TCO film and *R_GG_*, the corresponding to GML.

The total optical reflectance spectra were measured in the wavelength range from 300 to 1500 nm by using a UV/Visible/NIR Perkin-Elmer Lambda 1050 spectrophotometer equipped with a 6 mm integrating sphere accessory. From these measurements the weighted reflectance *R_p_*, defined as,

(2) $$R_{p}=\frac{\int_{400}^{1100}R_{hem}(\lambda )\times e(\lambda )\times d\lambda }{\int_{400}^{1100}e(\lambda )\times d\lambda }$$

was calculated to evaluate its anti-reflectance (AR) capability, where $R_{hem}(\lambda )$ is the hemispherical reflectance (total optical reflectance) as a function of the wavelength and *e*(*λ*) is the global spectral AM1.5G irradiance [18].

Specular transmittance spectra (*T*) of the fabricated TCEs deposited on glass were measured by using a three-detector module in the Perkin Elmer spectrophotometer. This measurement serves to determine the fraction of light that reaches the absorber of the solar device. Finally, optical transmission maps were obtained from the TCEs fabricated on glass by means of a home-made system combining a focused white-light lamp, an X-Y linear-positioner set, a pair of current preamplifiers, a reference photodiode and a pair of digital voltmeters. 

The system is controlled by specific software that returns an image where each point registered is associated with a specific transmittance value. Once the data is collected, it is represented in the form of a histogram where two peaks appear: one of them corresponds to the background transmittance (*T_B_*), and the other one is directly related to the combined transmittance of the sample plus the background (*T_SA_* + *T_B_*). *T_SA_* comprises the transmittance of both the substrate and the different TCEs (TCO and/or GML). Thus, knowing the transmittance of the substrate (*T_SUB_*), it is possible to calculate the amount of light transmitted by the different layers (*T_GG_* for the GML and *T_TCO_* for the TCO) as follows:

(3) $$T_{GG+TCO+SUB}=\frac{T_{GG+TCO+SUB+B}}{T_{B}}$$

(4) $$T_{GG+TCO}=\frac{T_{GG+TCO+SUB}}{T_{SUB}}$$

This measurement permits to verify the number of monolayers transferred and its effect on the transmittance of the whole TCE structure. In addition, the optical homogeneity of the TCE and the goodness of the graphene transferred can also be evaluated. 

Finally, a mapping of electrical conductance was carried out using non-contact and non-destructive commercial Onyx system from Das Nano Company, based on reflection-mode terahertz time-domain spectroscopy (THz-TDS) [19], where the measurable frequency range analyzed was from 0.1 to 5 THz. This patented system [20] provides a full-area map of conductance, resistance and other electrical parameters, related to 2D materials and thin films. The maps also show information about the homogeneity and quality of the deposition process. In addition, compared to other large-area methods, Onyx is capable to cover the gap between nano-scale and macro-scale methods. Its spatial resolution in the order of few hundreds of microns enables a fast characterization of large areas as opposed to microscopic methods.

## 3. Results and Discussion

The evaluation of the compatibility between the TCO and the GML was carried out in terms of the electrical and the optical properties of the whole structure. Table 1 shows the measured electrical sheet resistances of the TCEs based on AZO as TCO material, as function of both the number of GML and the configuration used (see Figure 1). For comparison, theoretical sheet resistances calculated from Equation (1) are included. For the calculation, measured R_TCO_ of 120 ± 10 Ω/sq, and *R_GG_* of 390 ± 10, 230 ± 30 and 120 ± 5 Ω/sq for one, two and three GML, respectively, were used. 

The contact resistivity of the Ti/Ag metallization was in the range of 3 to 10 mΩ·cm^2^. As it can be appreciated, the measured *R_TCE_* values differed considerably from the theoretical ones, especially in the case of configuration 2 where the TCO is sputtered on top of the transferred graphene. This suggests that GML might get damaged by the bombardment of the high-energy sputtered atoms during the sputtering process of the TCO thin film. This process could induce significant disorder, affecting the electrical performance of the GML [21,22]. 

On the other hand, the results of the *R_TCE_* in configuration 1 (graphene transferred onto sputtered TCO), summarized also on Table 1, showed a very slight improvement when the GML were added, in comparison to the bare AZO layer deposited on glass. However, these experimental values are still far from those predicted theoretically. This fact was attributed to a possible aluminum diffusion from the AZO into the graphene that would lead to the formation of different compounds, such as aluminum carbide, affecting the graphene order and hence, its electrical performance [23]. In addition, they also seemed to indicate that the aluminum diffusion into the GML affects their structure degrading their electrical performance. 

Therefore, another TCO material free of aluminum in its composition, such as ITO, was tested. This material was preferred because of its good conductivity and its optical transparency. The electrical performance of the ITO-based TCEs are summarized in Table 2; in this particular case, for the calculation, a measured value of *R_TCO_* is 85 ± 5 Ω/sq was used. 

The results from Table 2 reveal an electrical detriment using the configuration 2, not so dramatic than in the case of using AZO. Besides, the TCE in configuration 1 shows a sheet resistance of 55 ± 5 Ω/sq, which fits well the theoretical value of 50 Ω/sq for this stack and is much lower than the sheet resistance of the bare 80-nm-thick ITO thin film. This fact showed a clear evidence of the advantage of the hybrid TCEs fabricated by first depositing the TCO film and then transferring the graphene layers onto it. This also demonstrates that the transfer of several GML onto a TCO allows to enhance the electrical characteristics of the TCE, which fit well to those calculated modelling the hybrid TCE as a stack of two parallel resistances corresponding to each of the materials forming it. This result contrasts with previous reports on a single graphene monolayer onto ITO films where the sheet resistance is hardly enhanced [24,25], thus remarking the importance of having a stack of a sufficient number of GML for obtaining a hybrid structure of improved electrical characteristics. Furthermore, it provides some support to the formulated hypotheses regarding the damage to the graphene by the sputtering ions and the possible formation of aluminum carbide in case of contact with AZO or other aluminum-containing compounds. Taking all these considerations into account, from an electrical point of view, the structure of TCE considered as optimum was constituted by a stack of three GML transferred on top of an 80-nm-thick ITO film.

**Figure 3:**
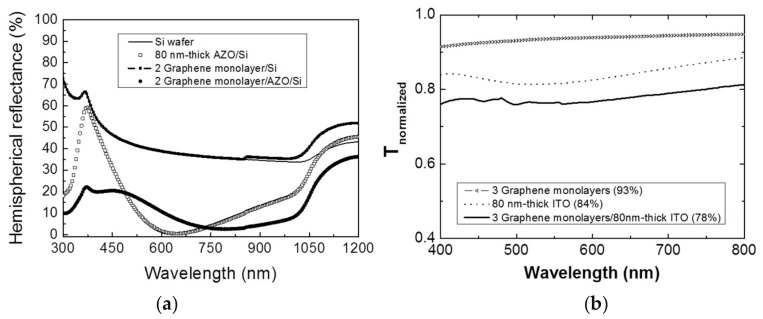
(**a**) Total (hemispherical) reflectance spectra in the visible range (300–875 nm) of a system of two GML stacked on top of a 80-nm-thick AZO film on silicon (fill circle symbol), a 80-nm-thick AZO film on silicon (open circle symbol), two GML on silicon (dot line), and the bare silicon wafer (straight line); (**b**) Normalized transmittance spectra in the visible range (400–800 nm) of a system of three GML stacked on top of a 80-nm-thick ITO film on a glass substrate (straight line) and a 80-nm-thick ITO film on glass (dot line) and 3 GML on glass (triangle symbol).

With regard to optical performance of hybrid TCEs developed, the optical characteristics were also addressed in both total reflection and transmission geometries. Thus, Figure 3a shows the hemispherical reflectance spectrum of the 2 GML/AZO/Si system compared to the spectra of the bare Si substrate, the 80-nm-thick AZO/Si structure and the 2 GML/Si stack. It should be noted here that the addition of graphene layers reduces the total weighted reflectance of the TCE from 12.7% to 10.8%, calculated using Equation (2), providing a better AR capability than the bare TCO layer. This superior AR capability when graphene is placed on top on TCO is considered as very promising to be incorporated as TCE in SHJ technology. On the other hand, Figure 3b shows the normalized transmittance spectra in the visible range (400–800 nm) of 80-nm-thick ITO layer on a glass substrate with and without a stack of three GML on top of the ITO film. The comparison of average transmittance data evidence an approximated 6% transmittance reduction, from 84% to 78%, as expected from the addition of the stack of three GML [26]. In addition, the average transmittance values calculated in the visible wavelength range were not affected by the TCE configuration.

**Figure 4:**
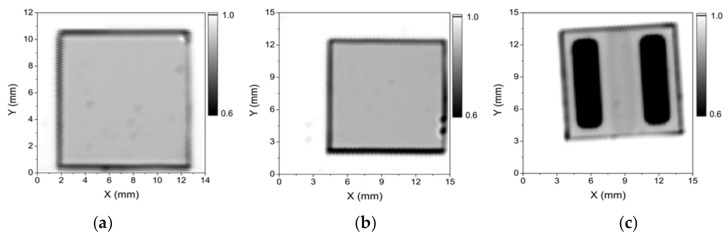
White-light optical-transmission maps of (**a**) 2 GML/glass; (**b**) 3 GML/glass; (**c**) TCE in configuration 2 (40-nm-thick AZO on 3 GML/glass, where the black zone corresponds to Ti/Ag metallization).

Finally, the white-light optical-transmission maps of the samples on glass substrates can reveal the number of GML transferred and the homogeneity of the transfer process. This technique is very useful because of its high sensitivity to small variations in thickness (either produced during the graphene growth or due to folds and wrinkles arising from the transfer process). Figure 4 shows the optical maps of (a) 2 GML, (b) 3 GML, both transferred on glass, and (c) 40-nm-thick AZO on 3 GML/glass system. The good homogeneity observed in the maps can be considered a proof of the quality of the graphene produced and the goodness and reproducibility of the automatic transfer process used.

Table 3 summarizes the white-light transmission values for the AZO-based TCEs in configuration 1 and 2.

Optical-transmission maps yielded white-light transmissions of 95% that corresponds to Figure 4a, and 93% that corresponds to Figure 4b. These values are consistent with the number of GML transferred, two and three, respectively [26].

On the other hand, regardless the TCE configuration, the white-light transmission values for the AZO-based TCEs are slightly lower than the bare AZO, as it was expected. In addition, no re-cover effect during thermal evaporation process was observed in Figure 4c. Thus, the optical properties do not depend neither on the TCO material nor the configuration used. Also the size of graphene layer can also be patterned to different geometries by electric fields [27,28,29]. 

**Figure 5:**
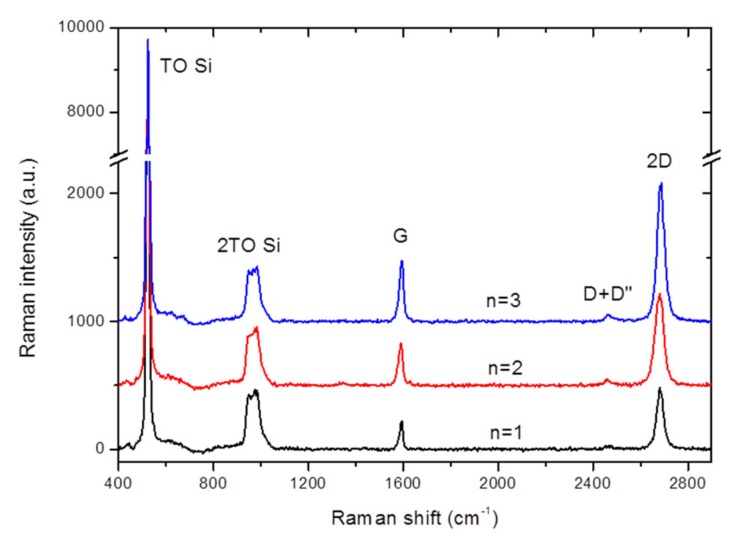
Raman spectra of a series of up to 3 GML stacked on top of the ITO/Si substrate system. The number of overlapping graphene layers (n) is indicated in each case. Spectra are vertically shifted for clarity.

The structural quality of the optimum hybrid ITO-based TCE, with configuration 1, was assessed. Figure 5 shows the Raman spectra of this sample. The Si substrate presented two peaks corresponding to the fundamental transverse optical (TO) phonon and its second order mode, 2TO. No Raman peaks were observed for the ITO film, although its presence is noted through a shift and broadening of the Si TO peak, as compared to the bare Si substrate [30]. The regions with different number of overlapping graphene layers, n = 1–3, were assessed. Graphene contributed with well-defined G and 2D Raman peaks and a negligible D + D’’ peak. In all three cases, there was no D peak and the ratio of 2D peak intensity to G peak intensity, I(2D)/I(G), was larger than 2, which is consistent with defect-free single-layer graphene. As n increased, both I(G) and I(2D) increased together, so that their ratio did not change significantly. This fact indicates that the transferred GML were randomly oriented, as expected. The Raman signature of the graphene also permits to estimate its carrier density [17], being the value similar to that extracted from the characteristics of a field-effect transistor [31]. In particular, the graphene monolayer presents a carrier density of 3.8 × 10^12^ cm^−2^.

**Figure 6:**
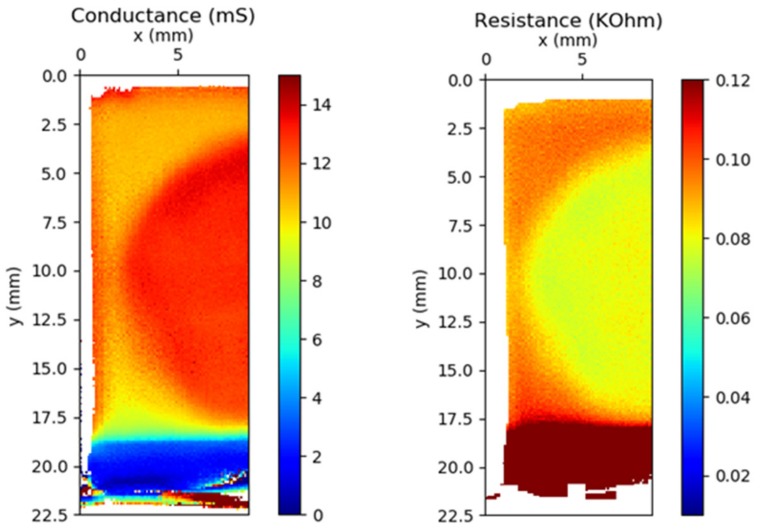
Conductance and resistance maps of 3 GML stacked transferred on top of the ITO/Si substrate system.

Finally, THz-TDS conductance and resistance maps acquired in reflection-mode for optimum hybrid ITO-based TCE deposited on Si substrate are pictures in Figure 6. The sample was positioned directly on the Onyx sample-holder without any pre-treatment. Scanning was performed in the x and y directions, with a window of 50 ps and 25 fs of temporal resolution. Measurement time was 10 min at 100 μm step-size resolution and a scanning speed of 15 mm^2^/min. The laboratory relative humidity was 50% and its temperature was 23 °C. All the electrical values were extracted using mathematical models detailed in [32]. From Figure 6a, a measured average conductance value of 13.12 mS (with a maximum of 14.03 mS) was obtained in the center of the sample, where the image provided a clear overview of the mm-scale uniformity; otherwise, a region of lower conductivity close to 10–12 mS was observed in the sample edge. This latter zone corresponds to the edge effects of GML transfer. In addition, the dark blue zone corresponds to the bare Si substrate (insulating), while the light blue region is related to ITO thin film that shows a conductance value close to 6 mS. To become an alternative and competitive TCE, sheet conductivities should be higher than ITO value, and hence, at least 10 mS were needed [19]. In comparison with the conductivity measured for 2 and 3 GML, 3 mS and 3.9 mS, respectively, a considerable increase was achieved by the hybrid TCE presented in this work. 

Regarding the resistance (Figure 6b), a central zone with 76.2 Ω/sq was calculated from conductance map, close to the sheet resistance of 55 ± 5 Ω/sq obtained from TLM measurements. This agreement confirms THz-TDS technique as very useful tool to characterize graphene-based TCEs. 

## 4. Conclusions

In this work, the compatibility of the graphene and the TCO deposition processes was analyzed with the aim of developing hybrid TCEs for silicon-based solar cell technology. The evaluation of TCEs made by coating the GMLs with a sputtered TCO layer indicated that this process severely affected the structure of graphene, leading to a significant detriment of sheet-resistance (up to values significantly higher than those of the bare GML). In contrast, the GML placed on top of a TCO led to TCEs with relatively good sheet resistances, particularly when the TCO material was ITO. This hybrid TCE showed sheet resistance values as low as 55 Ω/sq. These results suggest the convenience of avoiding aluminum in the TCO material, and hence, the choice of ITO is considered as suitable to achieve an optimum TCE structure. The in-depth electrical analysis using the THz-TDS technique led to average conductance values as high as 13.12 mS and a sheet resistance of 76.2 Ω/sq, improving the values of a common TCO. Optically, it was also verified that graphene transferred on TCO caused a total reflectance decrease, reaching values of weighted reflectance 2–3% lower than those obtained without graphene. All these results suggest the possibility of a noticeable improvement for TCEs by developing structures combining graphene with TCOs. Such hybrid TCEs would have optical and electrical properties favoring the absorption of more photons, hence leading to positive effects on the short circuit current, as well as lowering the cell series resistances. 

## Figures and Tables

**Table 1:** Theoretical and measured sheet resistances of the TCEs as function of the number of GML and the configuration used.

Number of GML	Theoretical *R_TCE_* (Ω/sq)	*R_TCE_* (Ω/sq) Configuration 1	*R_TCE_* (Ω/sq) Configuration 2
1	92	106 ± 5	2830 ± 30
2	79	127 ± 6	790 ± 80
3	60	116 ± 6	450 ± 50

**Table 2:** Theoretical and measured sheet resistances of the TCEs that incorporated a stack of three GML and ITO.

Number of GML	Theoretical *R_TCE_* (Ω/sq)	*R_TCE_* (Ω/sq) Configuration 1	*R_TCE_* (Ω/sq) Configuration 2
3	50	55 ± 5	156.5 ± 15

**Table 3:** Measured white-light transmission as function of both the number of GML and the TCE configuration used.

Number of GML	White-light T (%) Configuration 1	White-light T (%) Configuration 2	White-light T (%) Bare GML
0	86.0	95.0	-
1	84.4	-	97
2	81.7	92.2	95
3	80.9	89.4	93
